# Multi-Institutional Integration of Circulating miRNAs and Protein Tumor Markers for Early Lung Cancer Detection

**DOI:** 10.1016/j.jtocrr.2026.101051

**Published:** 2026-07-10

**Authors:** Ping-Han Hsieh, Ching-Yang Wu, Tsung-Ting Hsieh, Hung-Chih Lai, Shih-Sen Lin, Ko-Han Lee, Sheng-Hsiung Yang, Chih-Feng Chian, Chih-Tsung Wen, Lun-Che Chen, Pei-Hung Chang, Yu-Li Su, Yen-Liang Kuo, Pai-Chien Chou, Jen-Yu Hung, Yen-Jung Lu, Hung-Ling Chen, Ming-Hsuan Chou, Chia-Wei Liu, Yu-Chuan Chang, Jason Chia-Hsun Hsieh

**Affiliations:** a Department of Biomechatronics Engineering, National Taiwan University, Taipei City, Taiwan; b Pharus Diagnostics, Zhubei City, Hsinchu County, Taiwan; c Division of Thoracic Surgery, Department of Surgery, Linkou Chang Gung Memorial Hospital, Taoyuan, Taiwan; d Division of Hematology and Oncology, Department of Internal Medicine, Shin Kong Wu Ho-Su Memorial Hospital, Taipei, Taiwan; e Division of Chest Medicine, Department of Internal Medicine, Shin Kong Wu Ho-Su Memorial Hospital, Taipei, Taiwan; f Bioinformatics and Systems Biology Program, University of California San Diego, La Jolla, California; g Division of Pulmonary Medicine, Department of Internal Medicine, Mackay Memorial Hospital, Taipei, Taiwan; h Division of Pulmonary and Critical Care, Department of Internal Medicine, Tri-Service General Hospital, Taipei, Taiwan; i Division of Thoracic Surgery, Department of Surgery, New Taipei City Tucheng Hospital, New Taipei City, Taiwan; j Division of Pulmonology Medicine, Department of Internal Medicine, National Taiwan University Hospital, Hsin-Chu Branch, Hsinchu, Taiwan; k Division of Hematology-Oncology, Department of Internal Medicine, Keelung Chang Gung Memorial Hospital, Keelung, Taiwan; l Division of Hematology-Oncology, Department of Internal Medicine, Kaohsiung Chang Gung Memorial Hospital, Kaohsiung, Taiwan; m Division of Chest Medicine, Department of Internal Medicine, Fu Jen Catholic University Hospital, Fu Jen Catholic University, New Taipei City 243089, Taiwan; n School of Medicine, College of Medicine, Fu Jen Catholic University, New Taipei City, Taiwan; o Division of Pulmonary Medicine, Department of Internal Medicine, Taipei Medical University Hospital, Taipei, Taiwan; p Division of Pulmonary and Critical Care, Department of Internal Medicine, Kaohsiung Medical University Chung-Ho Memorial Hospital, Kaohsiung, Taiwan; q College of Medicine, Chang Gung University, Taoyuan, Taiwan; r Division of Hematology-Oncology, Department of Internal Medicine, Linkou Chang Gung Memorial Hospital, Taoyuan, Taiwan; s School of Medicine, National Tsing Hua University, Hsinchu, Taiwan

**Keywords:** Lung cancer, Early cancer detection, Circulating miRNAs, Diagnostic biomarkers, Multi-institutional study

## Abstract

**Introduction:**

Early detection of lung cancer is essential for improving survival rates. However, previously reported diagnostic biomarkers based on circulating microRNAs (cfmiRNAs) are often hindered by single-institutional biases and a lack of reproducibility. This study aimed to identify and verify a robust cfmiRNA-based diagnostic signature integrated with protein tumor markers using a large-scale, multi-institutional cohort.

**Methods:**

A multi-institutional observational study was conducted across 11 hospitals in Taiwan between 2024 and 2025. Plasma samples were collected from 752 participants, including 255 preoperative patients with lung cancer and 497 healthy controls. To mitigate site-specific effects and confounding factors, candidate cfmiRNAs were identified through stability selection with Monte Carlo sampling. A multiomics classification model was developed by integrating the miRNA panel with established protein markers (CEA, CA19-9, SCC, and Cyfra21-1).

**Results:**

A 15-miRNA diagnostic biosignature, functionally linked to tumor progression and cellular stress-related pathways, was identified. The final integrated multiomics model achieved an area under the ROC curve of 84% (95% confidence interval: 76–91) in an independent verification data set. At a fixed specificity of 80%, the cancer detection model reported a sensitivity of 73%. The multi-institutional approach provided a more generalized assessment of biomarker performance than localized cohorts.

**Conclusions:**

Our findings demonstrate the potential of multiomics integration in overcoming institution-specific biases and provide the necessary groundwork for lung cancer detection in an Asian population.

## Introduction

Early detection of lung cancer is crucial for improving patient prognosis and reducing mortality, as the disease is often diagnosed at an advanced stage with limited therapeutic options.^1^ Current lung cancer screening strategies rely heavily on low-dose computed tomography (LDCT) or computed tomography (CT) scans, which have proven effective in facilitating earlier diagnosis and lowering mortality among high-risk individuals. Randomized controlled trials consistently demonstrate the effectiveness of LDCT screening for lung cancer. The National Lung Screening Trial reported an approximately 20% reduction in lung cancer mortality^2^; the United Kingdom Lung Cancer Screening Trial observed a 16% reduction^3^; and the Dutch–Belgian Lung Cancer Screening Trial (NELSON) reported a more substantial, sex-specific reduction of 24% in men and 33% in women.^4^ Collectively, these studies report that early detection of lung cancer can improve patient outcomes and reduce mortality.

Despite its effectiveness, LDCT/CT screening faces a major challenge because of a high rate of false-positive results. For example, the National Lung Screening Trial reported a positive predictive value of only 3.6%, whereas the NELSON trial reported a higher, but still limited value of 43.5%. This high false-positive rate contributes to overdiagnosis, resulting in unnecessary follow-up imaging and invasive procedures, which in turn increase patient anxiety and health care costs.^5^^,^^6^ These limitations highlight the need for complementary screening approaches that can collectively enhance diagnostic specificity and overall screening efficacy. Among many molecular biomarkers and assays, microRNAs (miRNAs) have emerged as promising biomarkers for early cancer detection. These small noncoding RNA molecules (18–25 nucleotides) play crucial roles in gene regulation^7^^,^^8^ and are highly stable in various biofluids due to encapsulation in exosomes or association with RNA-binding proteins.^9^^,^^10^ They are readily detectable in noninvasive specimens, such as plasma or serum, and exhibit disease-specific dysregulation that reflects early pathological changes in tumor development. Together, these characteristics make miRNAs ideal candidates for noninvasive early cancer detection.^11^, ^12^, ^13^

Notably, alterations in miRNA expression are often detectable in early-stage disease, as reported across multiple cancer types, including lung cancer.^14^, ^15^, ^16^, ^17^, ^18^ These changes can reflect early molecular and regulatory events in tumor development, often before clinical symptoms emerge or lesions become detectable. Therefore, miRNAs are increasingly recognized for their applicability in early lung cancer screening, motivating efforts to develop panels that combine multiple miRNAs to improve diagnostic efficacy. For instance, Ying et al.^19^ established a five-miRNA panel (let-7a-5p, miR-1-3p, miR-1291, miR-214-3p, and miR-375) for detecting NSCLC. Abdipourbozorgbaghi et al.^18^ created distinct panels for lung adenocarcinoma and lung squamous cell carcinoma. Their lung adenocarcinoma panel consists of seven miRNAs (miR-9-3p, miR-96-5p, miR-147b-3p, miR-196a-5p, miR-708-3p, miR-708-5p, and miR-4652-5p), whereas the lung squamous cell carcinoma panel includes nine miRNAs (miR-130b-3p, miR-269-3p, miR-301a-5p, miR-301b-5p, miR-744-3p, miR-760, miR-767-5p, miR-4652-5p, and miR-6499-3p).^18^

Despite the promising potential of miRNAs as noninvasive diagnostic biomarkers, several challenges remain. A major limitation is the considerable inconsistency in the miRNA biomarker panels reported across different studies and populations, reflecting variability in patient cohorts and analytical methods, which may compromise the transferability of the proposed biomarkers and models.^20^, ^21^, ^22^ These challenges underscore the need for population-specific investigations and the development of miRNA panels tailored to the genetic background and environmental exposures of the target cohort.

## Materials and Methods

### Study Population

Between May 5, 2024, and November 14, 2025, this study consecutively enrolled individuals aged 18 years or older who underwent LDCT or CT scans for lung cancer screening across 11 hospitals in Taiwan, including Fu Jen Catholic University Hospital, Kaohsiung Chang Gung Memorial Hospital, Kaohsiung Medical University Chung-Ho Memorial Hospital, Keelung Chang Gung Memorial Hospital, Linkou Chang Gung Memorial Hospital, Mackay Memorial Hospital (Taipei Branch), Shin Kong Wu Ho-Su Memorial Hospital, Taipei Medical University Hospital, Tri-Service General Hospital, New Taipei City Tucheng Hospital, and National Taiwan University Hospital (Hsin-hu Branch). Detailed study protocols and registration information are available at https://clinicaltrials.gov/study/NCT07182214.

To minimize confounding biological factors, participants were excluded from this study if they had (1) a prior history of cancer diagnosis, (2) receipt of a nonautologous blood transfusion within 2 months before enrollment, (3) concurrent participation in an interventional clinical trial, (4) receipt of gene therapy within 1 year before enrollment, (5) administration of any vaccination within 3 months before blood collection, (6) current pregnancy, or (7) the presence of severe uncontrolled chronic lung conditions, any psychiatric conditions, or laboratory abnormalities that, in the principal investigator’s judgment, could confound data interpretation or pose unacceptable risks to the participant.

**Figure 1:**
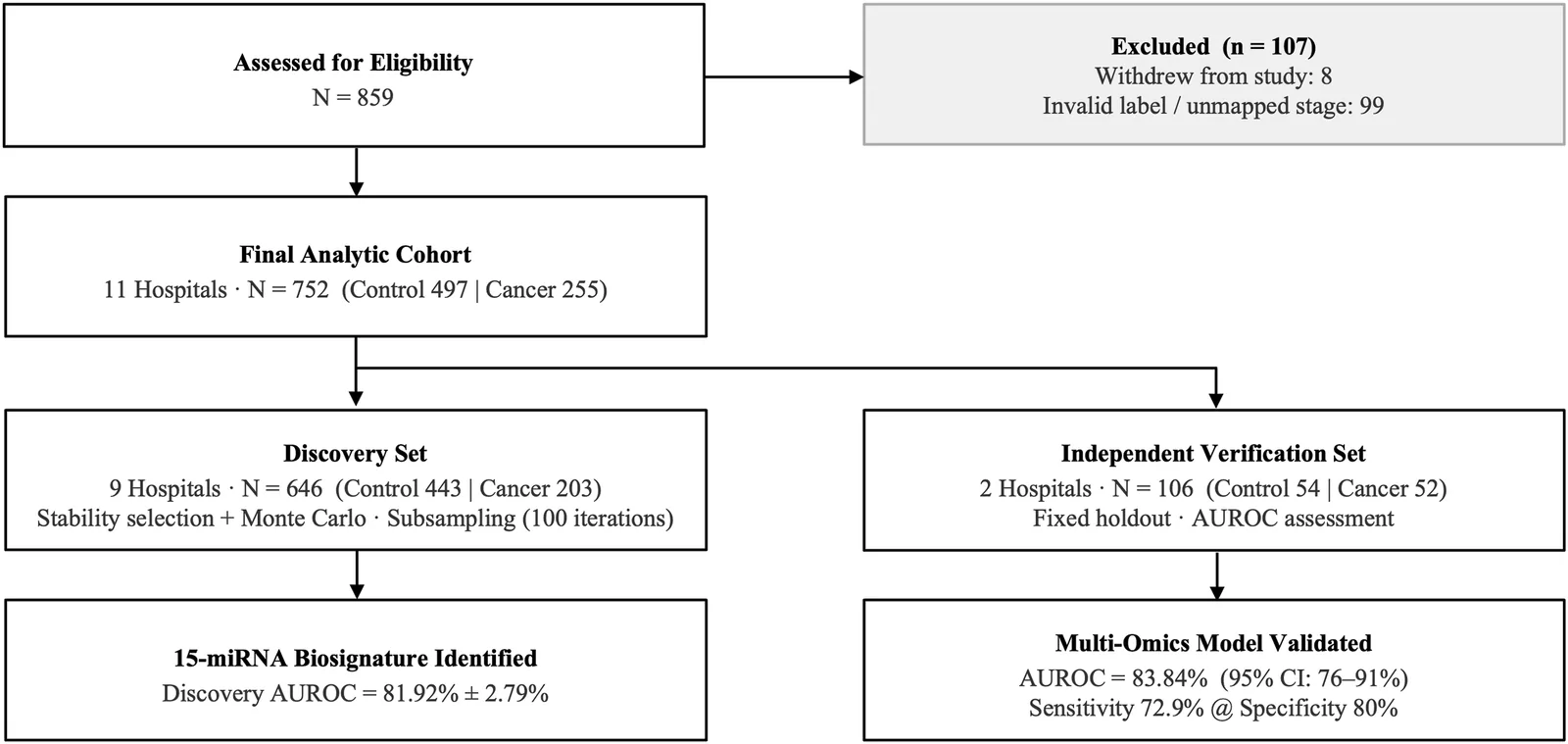
CONSORT-style participant flow diagram. A total of 859 participants were assessed for eligibility in the prospective study (NCT07182214; May 2024–November 2025). Of these, 107 were excluded (8 withdrew from the study and 99 had unclassifiable diagnoses or unassigned pathological stage). The final analytic cohort comprised 752 participants, which was divided into a Discovery Set (9 hospitals, n = 646) and an Independent Verification Set (2 hospitals, n = 106).

Participants were categorized into two primary classification groups based on their pathological and clinical diagnoses. Patients with lung nodules or masses of more than or equal to 6 mm detected on LDCT/CT imaging who were scheduled for a medical operation further underwent pathological examination of the surgically resected specimens. Among these patients, those with pathologically confirmed malignant disease were designated as the *cancer/case* group. Conversely, the *benign/control* group was composed of two distinct sets of participants. The benign set included patients from the surgical group whose resected specimens were pathologically confirmed as benign lesions, whereas the control set consisted of individuals with unremarkable findings on LDCT/CT scans, as determined by physicians (Fig. 1).

### Specimen Collection

Peripheral venous blood samples, clinical diagnostic data, and demographic information were collected from all participants. For patients undergoing surgery, blood samples were obtained before the scheduled procedure or treatment without fasting requirements to minimize perioperative fluctuations in biomarker levels. For participants in the control group, blood was collected within 2 months of their last LDCT/CT scan, also without fasting requirements.

From each participant, one ethylenediaminetetraacetic acid (EDTA) tube (approximately 10 mL) and one serum separator tube (SST) (approximately 8 mL) of peripheral blood were collected. The 10 mL EDTA sample was used for cell-free miRNA (cfmiRNA) profiling, whereas the SST sample was used to measure established protein tumor markers, including carcinoembryonic antigen (CEA), carbohydrate antigen 19–9 (CA19–9), squamous cell carcinoma antigen (SCC), and cytokeratin 19 fragment (Cyfra21-1). Protein tumor markers were quantified primarily at the hospital’s clinical laboratory. In cases where the hospital laboratory was unavailable, measurements were performed in collaboration with eRSSbyCL using a Chemiluminescent Microparticle Immunoassay. These measurements provided supporting clinical evidence for the hypothesis investigated in this observational study. All blood samples were gently inverted 10 times immediately after collection. Plasma separation was performed within 1 hour of blood collection using a two-step differential centrifugation protocol: an initial spin at 1200 × g for 10 minutes to pellet cells, followed by a high-speed spin at 12,000 × g for 10 minutes to remove residual cellular debris. After the initial centrifugation, the supernatant was assessed for hemolysis using a hemolysis chart; samples with a color indicating a hemoglobin concentration below 50 mg/dL were classified as non-hemolyzed and subsequently processed for miRNA isolation. The resulting plasma was aliquoted and immediately stored at –80°C until further analysis.

### cfmiRNA Isolation, Library Preparation, Sequencing and Quantification

For cfmiRNA isolation, plasma samples were processed using the miRNeasy Serum/Plasma Advanced Kit (Qiagen) on the QIAcube Connect (Qiagen) automated platform, following the manufacturer’s protocol. miRNA libraries were then constructed using the QIAseq miRNA Library Kit (Qiagen) in combination with the QIAseq miRNA UDI Index Kits (Unique Dual Index) (Qiagen). Library concentrations were assessed using a Qubit fluorometer (Thermo Fisher Scientific). Individual libraries were pooled and sequenced on an Illumina NextSeq 550 (Illumina, Inc.) platform, generating single-end 72 bp reads. Raw sequencing reads were demultiplexed and subjected to quality control and quantification using the QIAGEN RNA-seq Analysis Portal (Qiagen). For annotation and quantification, sequences were mapped to miRBase v22.^23^ Raw miRNA count data were normalized using upper-quartile normalization with the 96th percentile (0.96 quantile) as the scaling factor. This method was chosen because it mitigates the impact of highly expressed miRNAs and provides robust adjustment for variations in library composition and sequencing depth. Moreover, it provides a transferable scaling factor, allowing the normalization to be applied to external validation data without recalculating statistics used for normalization based on the distribution of new cohorts. In addition, it can be directly applied to any number of samples, including single samples, facilitating the potential translation to clinical applications.^24^ Samples with missing values in clinical metadata or established tumor markers were excluded from the downstream analysis.

### Biomarkers Discovery and Verification

To identify potential diagnostic biomarkers, we adopted a nested Monte Carlo subsampling framework. The discovery data set was repeatedly split 100 times into training (70%) and testing (30%) sets without replacement using stratified sampling to preserve class proportions in the population. Potential diagnostic miRNA biomarkers were identified using stability selection.^25^ In each resampling iteration, an eXtreme Gradient Boosting (XGBoost) model^26^ was fitted to each subsampled training data set. miRNAs with non-zero feature-importance scores in more than 40% of the iterations were identified as potential diagnostic biomarkers. This multivariate strategy allows all miRNAs to be modeled jointly while naturally accounting for correlations among features. Coupled with repeated subsampling, this procedure ensures that the selected biomarkers are robust and stable. After feature selection, XGBoost was trained on the full discovery data set using the discovered biomarkers. To robustly evaluate the diagnostic efficacy of these biomarkers, the cohort was split to establish an independent verification data set for validating the classification model (see Table 1 for more details).

**Table 1:** Demographic Characteristics of the Participant Cohort Across All Collaborating Hospitals

Hospital	Group	N	Age (yr) mean ± SD	Male (%)	Smoker (%)	NSCLC (%)	Tumor Stage			
							0	I–II	III–IV	Unk
**All Participants**	**Case**	**255**	**59.9 ± 11.0**	**37.6**	**29.4**	**97.3**	**44**	**155**	**56**	**0**
	**Control**	**497**	**58.8 ± 10.8**	**47.5**	**25.6**	**—**	**—**	**—**	**—**	**—**
**Discovery Set — 9 Hospitals (n = 646)**										
Fu Jen Catholic University Hospital	Case	2	59.0 ± 2.8	100.0	0.0	100.0	1	1	0	0
	Control	31	54.5 ± 10.8	54.8	38.7	—	—	—	—	—
Kaohsiung Chang Gung Memorial Hospital	Case	4	62.2 ± 11.9	50.0	25.0	100.0	0	3	1	0
	Control	39	57.4 ± 11.8	41.0	20.5	—	—	—	—	—
Kaohsiung Medical University Chung-Ho Memorial Hospital	Case	2	71.0 ± 2.8	0.0	0.0	100.0	0	2	0	0
	Control	9	53.3 ± 12.6	88.9	44.4	—	—	—	—	—
Keelung Chang Gung Memorial Hospital	Case	2	50.0 ± 5.7	0.0	0.0	100.0	0	2	0	0
	Control	63	56.1 ± 8.8	44.4	31.7	—	—	—	—	—
Linkou Chang Gung Memorial Hospital	Case	104	58.0 ± 10.5	26.9	21.2	99.0	18	83	3	0
	Control	50	56.9 ± 12.4	50.0	30.0	—	—	—	—	—
Mackay Memorial Hospital (Taipei)	Case	33	65.6 ± 11.0	48.5	48.5	93.9	4	10	19	0
	Control	73	67.3 ± 11.0	54.8	20.5	—	—	—	—	—
Shin Kong Wu Ho-Su Memorial Hospital	Case	36	59.1 ± 10.1	47.2	47.2	88.9	1	25	10	0
	Control	83	54.0 ± 9.6	47.0	19.3	—	—	—	—	—
Taipei Medical University Hospital	Case	10	68.3 ± 13.6	70.0	70.0	80.0	1	3	6	0
	Control	2	65.0 ± 15.6	50.0	50.0	—	—	—	—	—
Tri-Service General Hospital	Case	10	65.3 ± 6.5	50.0	20.0	90.0	0	5	5	0
	Control	93	59.9 ± 8.0	40.9	21.5	—	—	—	—	—
**Verification Set — 2 Hospitals (n = 106)**										
New Taipei City Tucheng Hospital	Case	15	65.5 ± 9.1	33.3	33.3	100.0	1	6	8	0
	Control	49	62.5 ± 7.4	42.9	28.6	—	—	—	—	—
National Taiwan University Hospital Hsin-Chu Branch	Case	37	54.4 ± 9.9	37.8	13.5	97.3	18	14	0	5
	Control	5	52.4 ± 13.6	60.0	40.0	—	—	—	—	—

Tumor Stage 0: carcinoma in situ; I–II: early stage disease; III–IV: late-stage disease; Unk (Unknown): staging not available. NSCLC; includes adenocarcinoma, minimally invasive adenocarcinoma, squamous cell carcinoma, and adenosquamous carcinoma. ‘—' indicates not applicable for control participants. Smoker (%): proportion with any history of tobacco smoking (ever-smokers). Male (%): percentage of male participants. Age: mean ± SD.

### Ethics

The protocol for data collection was conducted in accordance with the Declaration of Helsinki and was reviewed and approved by the Institutional Review Board or Institutional Ethics Committee of all participating hospitals (see Supplementary Materials 2). All participants provided written informed consent before enrollment in accordance with the Declaration of Helsinki. The informed consent process was conducted by qualified clinical personnel at each participating hospital. Participants were informed of the study objectives, procedures, potential risks, and their right to withdraw at any time without affecting their medical care. All biospecimens were collected by qualified medical personnel following standard clinical procedures. To ensure the confidentiality and privacy of the participants, all clinical data and biological samples were de-identified before being transferred for laboratory analysis and statistical modeling, maintaining strict data protection throughout the research process.

## Results

### Limited Transferability of Literature-Reported Biomarker Panels

To evaluate the transferability of previously reported lung cancer miRNA biomarkers, we evaluated their predictive performance in our multi-institutional cohort using the panels described by Abdipourbozorgbaghi et al.^18^, Liao et al.,^27^ and Ying et al.^19^ The complete list of miRNA biomarkers is provided in Supplementary Table 1. To evaluate the predictive efficacy of previous research, we fit an XGBoost classification model to the discovery data sets using the same hyperparameters throughout this study based on the biomarker panel, and the performance was evaluated on the hold-out verification data set. Overall, the classification models based on previously identified biomarkers demonstrated limited predictive capability in our study, with area under the receiver operating characteristic curve (AUROC) values ranging from 51.69% to 64.83% and consistently low sensitivity. These results suggest poor transferability of these biomarker panels to the Taiwanese population (Supplementary Table 2).

### Biomarker Discovery and Predictive Performance

**Figure 2:**
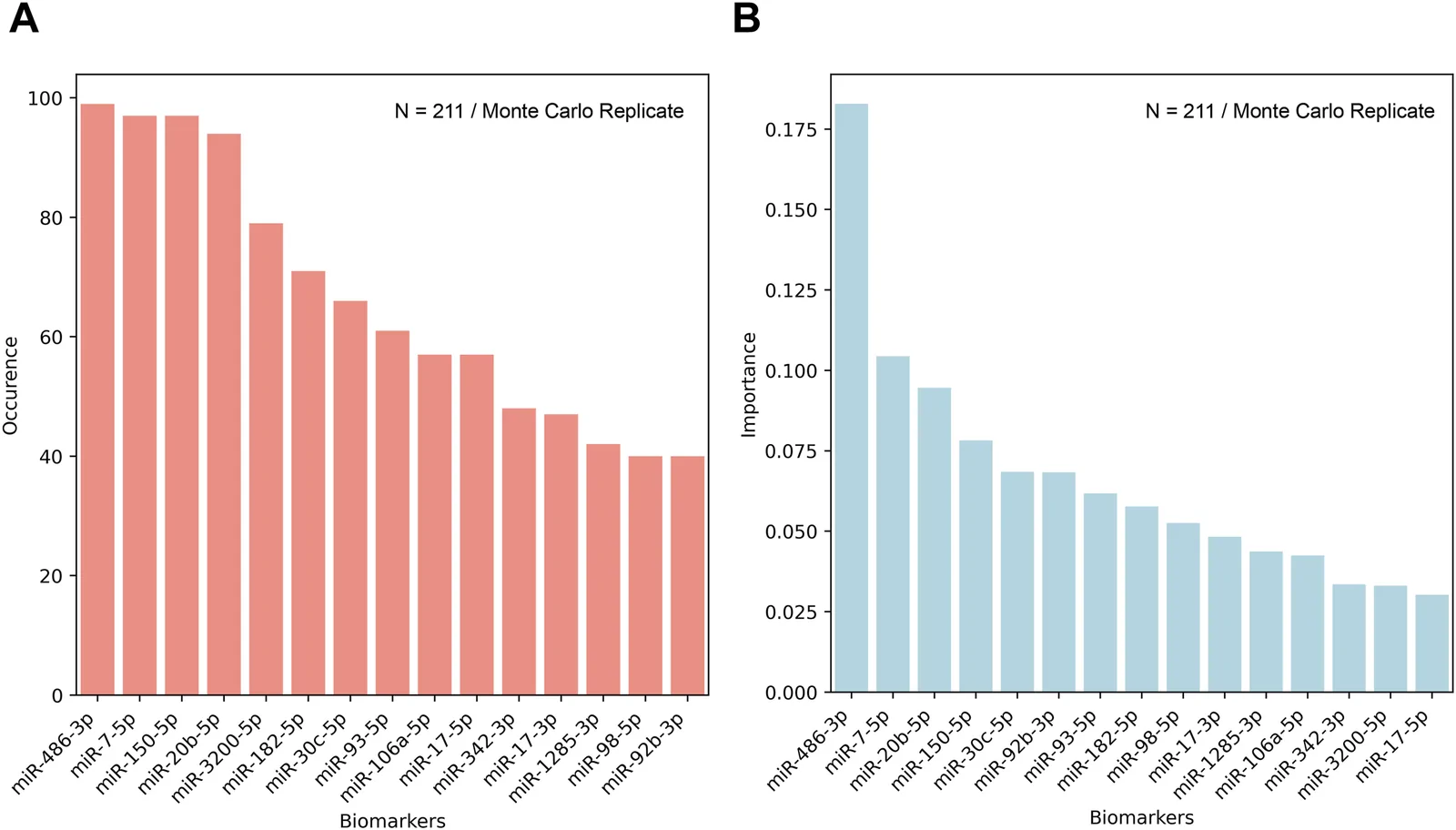
(*A*) Selection frequency of the potential miRNA biomarker across 100 Monte Carlo subsampling replicates using eXtreme Gradient Boosting (XGBoost) feature importance. (*B*) XGBoost derived feature importance of the identified miRNA biomarkers contributing to the classification model.

**Figure 3:**
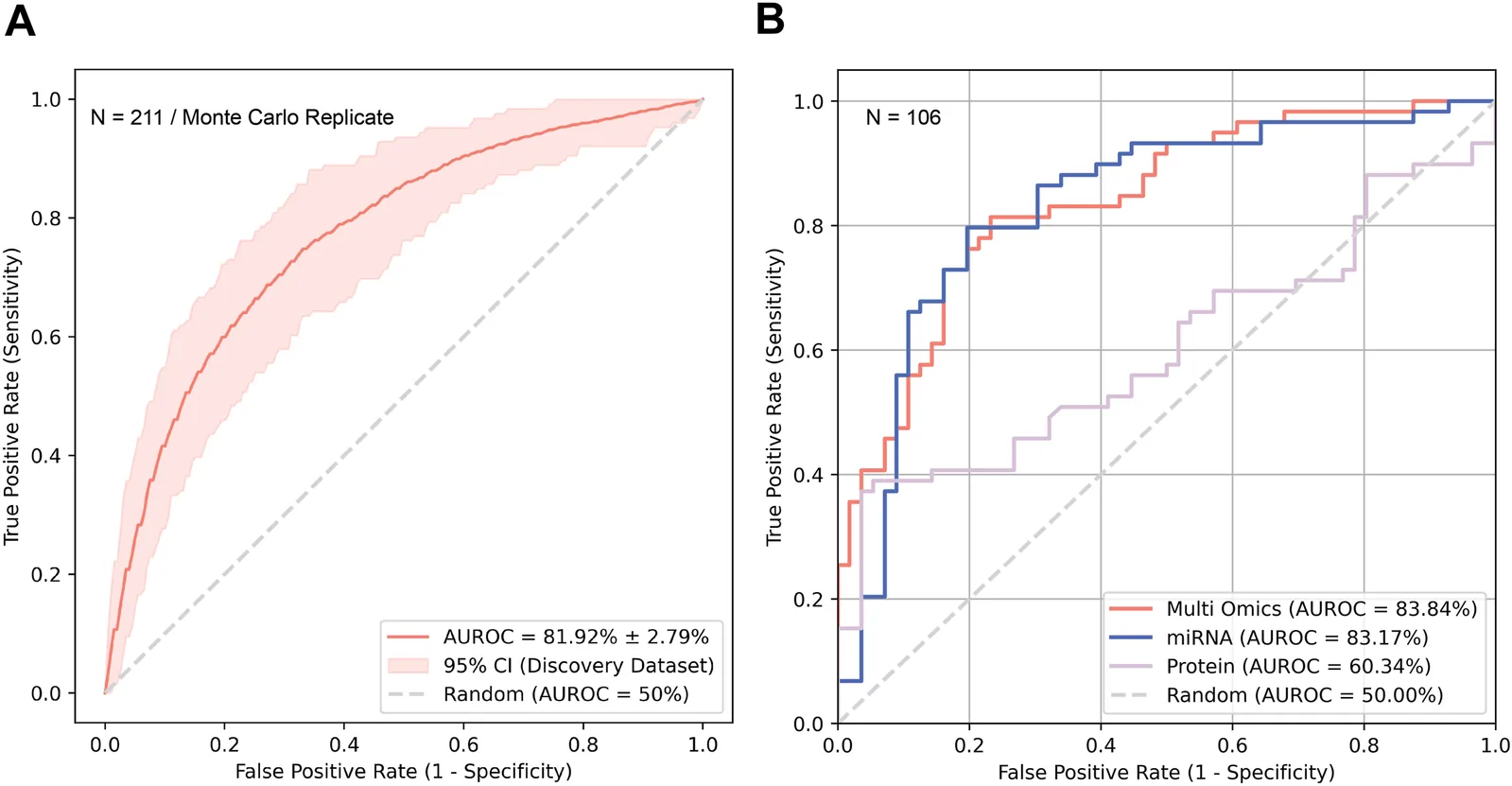
Predictive performance of the classification models. (*A*) Receiver operating characteristic (ROC) curves illustrating the predictive performance of the classification model across 100 Monte Carlo subsamples in the discovery data set (*B*) ROC curves illustrating the predictive performance of classification models trained using different feature sets in the independent validation data set.

Applying stability selection to Monte Carlo subsampled data sets enables the identification of candidate biomarkers. Using this approach, we analyzed 100 Monte Carlo sampled replicates and identified 15 potential miRNA biomarkers (miR-486-3p, miR-7-5p, miR-150-5p, miR-20b-5p, miR-3200-5p, miR-182-5p, miR-30c-5p, miR-93-5p, miR-106a-5p, miR-17-5p, miR-342-3p, miR-14-3p, miR-1285-3p, miR-98-5p, and miR-92b-3p). The selection frequency of each biomarker across the bootstrap replicates is shown in Figure 2*A*. Among the discovered biomarkers, miR-486-3p, miR-7-5p, miR-150-5p, and miR-20b-5p were the most stable, being selected in over 90% of the subsampled data sets; all 15 identified biomarkers were also consistently detected across all samples in the verification data set (Supplementary Fig. 1). The predictive performance of the diagnostic model based on the AUROC is shown in Figure 3*A*. The XGBoost classification model trained on the full discovery data set reported strong predictive performance based on the identified miRNA biomarkers, achieving an AUROC of 83.17% and an overall accuracy of 78.26% (Table 2). Moreover, the feature importance scores for the identified biomarkers generally followed the same tendency as their selection frequencies. (Fig. 2*B*).

**Table 2:** Predictive Performance Metrics (with 95% Confidence Intervals) of Proteins, miRNAs, and Multiomics Classification Models based on Verification Data set (Specificity Controlled at 80%)

Metric	Proteins	miRNAs	Multiomics
Accuracy	60.87% (51.87**–**69.87)	78.26% (70.65**–**85.87)	78.26% (70.65**–**85.87)
Sensitivity	40.68% (27.93**–**53.43)	72.88% (61.34**–**84.42)	72.88% (61.34**–**84.42)
PPV	70.59% (55.18**–**86.00)	82.69% (72.27**–**93.11)	82.69% (72.27**–**93.11)
NPV	56.79% (45.89**–**67.69)	74.60% (63.80**–**85.40)	74.60% (63.80**–**85.40)
AUROC	60.34% (49.93**–**70.75)	83.17% (75.60**–**90.74)	83.84% (76.41**–**91.27)
Control (N = 54), Case (N = 52)			

AUROC, area under the receiver operating characteristic curve; CI, confidence interval; NPV, negative predictive value; PPV, positive predictive value.

### Integration of Protein Tumor Markers Improves Predictive Efficacy

After the identification of potential miRNA biomarkers, we developed a classification model that integrates multiomics data to capture complementary molecular signatures. Specifically, we integrated the discovered miRNA biomarkers from the combined training data with four established protein tumor markers (CEA, CA19-9, SCC, and Cyfra21-1) to evaluate the performance of the integrated classification model, in comparison with the model built using the protein markers solely. The classification model based solely on protein tumor markers performed worse than the model using the identified miRNA biomarkers. However, the protein tumor marker-based model achieved improved performance in the late-stage (stage III and IV) cohort compared with the miRNA-only model, whereas its contribution was less pronounced in early-stage (stage I, II, and carcinoma in situ) disease (Supplementary Fig. 2). These findings suggest that protein tumor markers and miRNA biomarkers may capture complementary aspects of tumor biology at different stages of disease progression.

Although integrating both molecular modalities does not substantially improve AUROC compared with using miRNA biomarkers alone, the integrative model achieves higher sensitivity when controlling for a very low false discovery rate, and the false-positive rate increases sharply once sensitivity exceeds 40%. Interestingly, among the 10 false-positive LDCT cases available in our verification cohort, three were correctly classified as controls by the multiomic model. Although limited by sample size, this highlights the potential of the integrative model to complement low-precision LDCT screening in early cancer detection (Table 1 and Fig. 3*B*).

### Molecular Feature Contributions to Multiomics Model

To gain insight into the relative contribution of each molecular feature to the model predictions, we applied SHapley Additive exPlanations (SHAP) analysis.^28^ This approach quantifies how each miRNA biomarker and protein tumor marker collectively influence the model output, highlighting the key molecular signatures driving classification and providing interpretability for the combined multiomics model.

**Figure 4:**
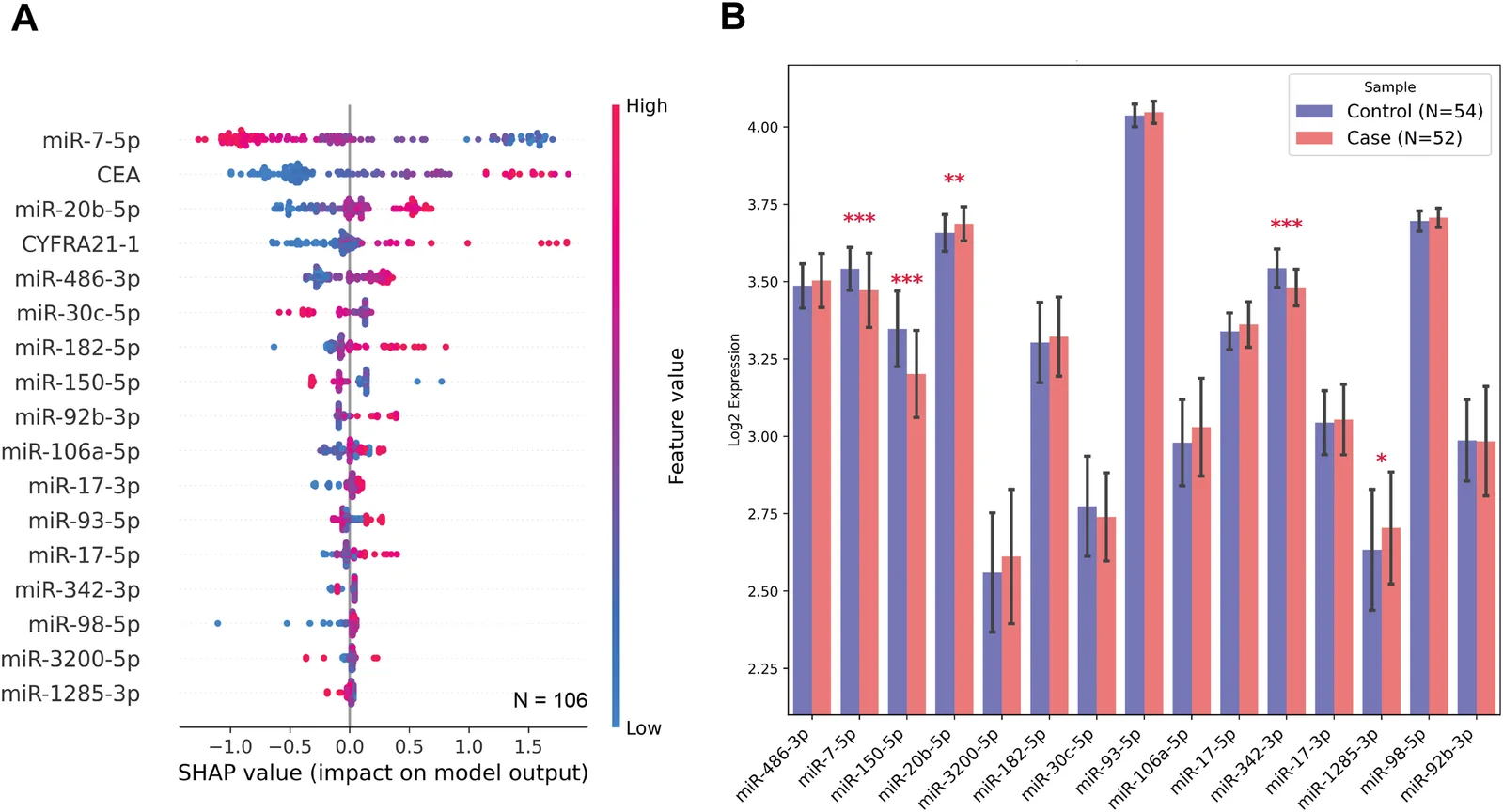
(*A*) SHapley Additive exPlanations (SHAP) analysis showing the contribution of miRNA and protein biomarkers to the multiomics prediction model. High SHAP values indicate features driving the prediction toward a positive prediction. (*B*) Box plots of biomarker expression in malignant cancer samples (case) and healthy controls. Statistical significance of Welch’s t-test between the two distributions is indicated by ∗ (*t*-test *p* ≤ 0.05), ∗∗ (*t*-test *p* ≤ 0.01) and ∗∗∗ (*t*-test *p* ≤ 0.001)

SHAP analysis revealed several molecular features that strongly influenced the predictions of the model (Fig. 4*A*). Among the investigated molecular features, upregulation of miR-20b-5p, CEA, and Cyfra21-1 was strongly associated with cancer cases. In contrast, upregulation of miR-7-5p, miR-150-5p, and miR-342-3p was associated with control samples. Although Welch’s t-test suggested that only miR-7-5p, miR-150-5p, miR-20b-5p, miR-342-3p, and miR-1285-3p showed significant differences between malignant cancer samples and healthy controls (Fig. 4*B*), SHAP analysis enabled us to evaluate the combinatory impact of multiple biomarkers simultaneously, capturing combinatory interactions and contributions between biomarkers that cannot be observed in univariate tests.

## Discussion

To our knowledge, this study represents the first multi-institutional analysis combining cfmiRNA and protein tumor markers for lung cancer detection in a Taiwanese cohort. Although most studies collect samples from only one or a few hospitals and randomly split the data into training and validation sets, we argue that this approach cannot fully account for site-specific biases, which may inflate performance and limit the generalizability of the identified biomarkers. To address this, we collected samples from 11 independent hospitals. This enables a robust evaluation of model performance and ensures that the classification model does not exploit site-specific biases. To further address potential confounding effects, we applied a Monte Carlo upsampling strategy combined with stability selection, enabling the identification of biomarkers that are more likely to be robust across institutions.

An important observation from this study is the limited transferability of several previously reported miRNA biomarker panels to our Taiwanese cohort. Although our objective was not to reproduce these previously published signatures, their reduced performance highlights the challenges of transferring biomarker panels across populations. This limitation may reflect differences in genetic background, environmental exposures, cohort composition, and sample processing protocols. For example, the signatures reported by Abdipourbozorgbaghi et al.^18^ and Liao et al.^27^ were developed using Swiss and United States cohorts, respectively, whereas Ying et al.^19^ used serum samples rather than the plasma samples analyzed in this study. These findings underscore the importance of evaluating biomarker transferability across populations and suggest that population-specific factors may substantially influence the performance of miRNA-based diagnostic signatures.

By combining multi-institutional training data, we derived a panel of 10 miRNA biomarkers, three of which were also identified in the panels derived from individual hospitals. These biomarkers have reported roles in tumor progression, cell-cycle regulation, apoptosis, cellular stress-related pathways, and oncogenic pathways; their contributions to the classification model closely reflect these established biological functions. Among these, miR-20b-5p has been reported to drive NSCLC growth through the Wnt/β-catenin signaling pathway.^29^ In contrast, tumor suppressor miRNAs such as miR-7-5p, miR-150-5p, and miR-342-3p have been shown to inhibit cell proliferation and metastasis through regulation of oncogenic signaling pathways, including PAK2, NOVA2, and HMGA2.^30^, ^31^, ^32^, ^33^, ^34^, ^35^, ^36^ Finally, miR-1285-3p, despite limited functional characterization, has been repeatedly identified as a potential diagnostic biomarker in lung cancer.^30^

We further investigated multiomics data integration by combining the miRNA signature with established protein tumor markers (CEA, CA19-9, SCC, and Cyfra21-1), which modestly improved predictive performance. This result demonstrates that a multimodal approach captures complementary aspects of tumor biology. This finding is consistent with previous research indicating that circulating miRNAs are dysregulated early in tumor development and may serve as sensitive, minimally invasive indicators of underlying pathological and regulatory changes.^11^^,^^13^ In contrast, established protein tumor markers, such as CEA and Cyfra21-1, typically correlate with tumor burden, host response, or metastatic progression and therefore tend to show higher specificity only in advanced disease, resulting in poorer sensitivity for early-stage detection.^37^, ^38^, ^39^ The ensemble model achieved moderate generalizability (AUROC = 83.84%) on the independent verification data set. Although these biomarkers show promising potential for early lung cancer detection, the overall predictive performance remains moderate, indicating that further optimization and validation are required to evaluate their robustness and clinical applicability across diverse populations.

Notably, because our cohort was specifically designed to address site-specific biases within Taiwan, the applicability of the identified miRNA panel to non-Taiwanese or ethnically diverse populations remains to be established. In line with the limited transferability observed for previously reported miRNA signatures in our cohort, these findings suggest that population-specific genetic backgrounds, environmental exposures, and technical factors may influence biomarker performance. This work represents an initial step toward establishing a robust, miRNA-based, noninvasive screening platform for early lung cancer detection within the Taiwanese population. Future studies should focus on expanding the integration of multi-modal molecular data to further enhance predictive power and complement current standard imaging approaches, such as LDCT/CT. Ultimately, large-scale prospective validation studies will be required to fully evaluate the clinical applicability and readiness of these biomarkers for population-level screening.

## CrediT Author Contribution Statement

**Ping-Han Hsieh:** Conceptualization, Formal analysis, Methodology, Software, Visualization, Writing – original draft.

**Ching-Yang Wu:** Conceptualization, Data curation, Resources, Writing – review & editing.

**Tsung-Ting Hsieh:** Formal analysis, Methodology, Software, Visualization.

**Hung-Chih Lai:** Data curation, Resources, Writing – review & editing.

**Shih-Sen Lin:** Data curation, Resources, Writing – review & editing.

**Ko-Han Lee:** Writing – original draft.

**Sheng-Hsiung Yang:** Data curation, Resources, Writing – review & editing.

**Chih-Feng Chian:** Data curation, Resources, Writing – review & editing.

**Chih-Tsung Wen:** Data curation, Resources, Writing – review & editing.

**Lun-Che Chen:** Data curation, Resources, Writing – review & editing.

**Pei-Hung Chang:** Data curation, Resources, Writing – review & editing.

**Yu-Li Su:** Data curation, Resources, Writing – review & editing.

**Yen-Liang Kuo:** Data curation, Resources, Writing – review & editing.

**Pai-Chien Chou:** Data curation, Resources, Writing – review & editing.

**Jen-Yu Hung:** Data curation, Resources, Writing – review & editing.

**Yen-Jung Lu:** Investigation.

**Hung-Ling Chen:** Investigation.

**Ming-Hsuan Chou:** Investigation.

**Chia-Wei Liu:** Investigation.

**Yu-Chuan Chang:** Conceptualization, Formal analysis, Methodology, Project administration, Resources, Software, Supervision, Writing – original draft.

**Jason Chia-Hsun Hsieh:** Conceptualization, Methodology, Project administration, Resources, Supervision, Writing – review & editing.

## Declaration of Generative AI and AI-Assisted Technologies in the Writing Process

During the preparation of this work, the author(s) used Gemini 3 Pro to improve readability and language. After using this tool/service, the author(s) reviewed and edited the content as needed and take(s) full responsibility for the content of the publication.

## Disclosure

T.-T.H., Y.-H.L., H.-L.C., M.-H.C., C.-W.L., Y.-C.C., are employees of Pharus Diagnostics (Pharus Inc.), and P.-H.H. was employed by the company from June 2024 to June 2025. During their employment, these authors received financial support in the form of salaries, and K.-H.L. received financial support in the form of consulting fees. T.-T.H., Y.-H.L., H.-L.C., M.-H.C., C.-W.L., and Y.-C.C. hold stock or stock options in Pharus Diagnostics. This research utilizes technologies and methodologies licensed by Pharus Diagnostics. A patent application is planned regarding diagnostic biomarkers and a classification algorithm utilizing circulating miRNAs and protein markers for the early detection of lung cancer. The aforementioned employed authors may receive related benefits from the patent. Furthermore, Pharus Diagnostics also provided the necessary laboratory instrumentation, computational infrastructure, and financial coverage for the article processing charges. The remaining authors declare no potential conflicts of interest.
