# Supplementary material for: Multi-Institutional Integration of Circulating miRNAs and Protein Tumor Markers for Early Lung Cancer Detection
Source: JTO Clin Res Rep. 2026 Jul 10;7(10):101051. doi: 10.1016/j.jtocrr.2026.101051 (PMC13584035; doi:10.1016/j.jtocrr.2026.101051)
Supplement: Supplementary file 1 [file mmc1.pdf]

Multi-Institutional Integration of miRNAs and Protein Tumor Markers for Early Lung Cancer Detection

Supplementary Materials

Table S1: miRNA panels reported by previous studies

Study	miRNA Panel
Abdipourbozorgbaghi et al. (LUSC) [1]	miR-130b-3p, miR-269-3p, miR-301a-5p, miR-301b-5p, miR-744-3p, miR-760, miR-767-5p, miR-4652-5p, miR-6499-3p
Abdipourbozorgbaghi et al. (LUAD) [1]	miR-9-3p, miR-96-5p, miR-147b-3p, miR-196a-5p, miR-708-3p, miR-708-5p, miR-4652-5p
Liao et al. [2]	miR-31-5p, miR-210-3p, miR-21-5p
Ying et al. [3]	let-7a-5p, miR-1-3p, miR-214-3p, miR-1291, miR-375-5p, miR-375-3p

Table S2: Predictive Performance Metrics (with 95% Confidence Intervals) of Proteins, miRNAs, and Multi-omics Classification Models based on Verification Dataset (Specificity Controlled at 0.80)

Metrics	Abdipourbozorgbaghi et al. (LUSC)	Abdipourbozorgbaghi et al. (LUAD)	Liao et al.	Ying et al.
Accuracy	45.22% (36.04-54.40)	57.39% (48.27-66.51)	53.04% (43.84-62.24)	59.13% (50.07-68.19)
Sensitivity	11.86% (3.47-20.25)	35.59% (23.16-48.02)	27.12% (15.58-38.66)	40.68% (27.93-53.43)
PPV	38.89% (16.63-61.15)	65.62% (49.09-82.15)	59.26% (40.72-77.80)	66.67% (51.18-82.16)
NPV	46.39% (36.34-56.44)	54.22% (43.39-65.05)	51.14% (40.58-61.70)	55.70% (44.64-66.76)
AUROC	51.69% (41.01-62.37)	64.83% (54.73-74.93)	60.50% (50.10-70.90)	58.81% (48.33-69.29)

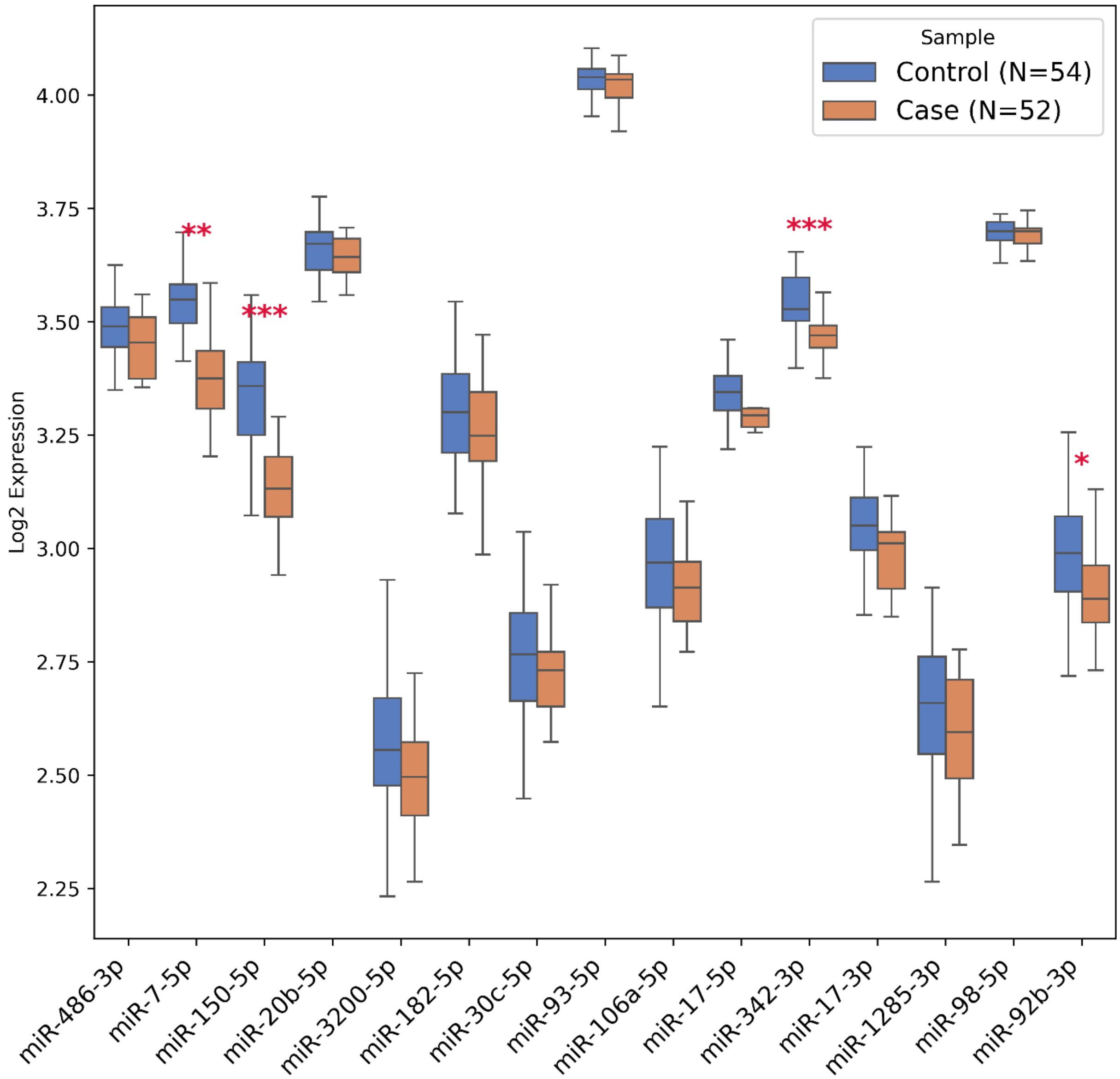

Supplementary Figure 1. Distribution of the Identified Biomarkers in the Verification Dataset. The results show that all identified biomarkers were consistently expressed across samples in the verification dataset.

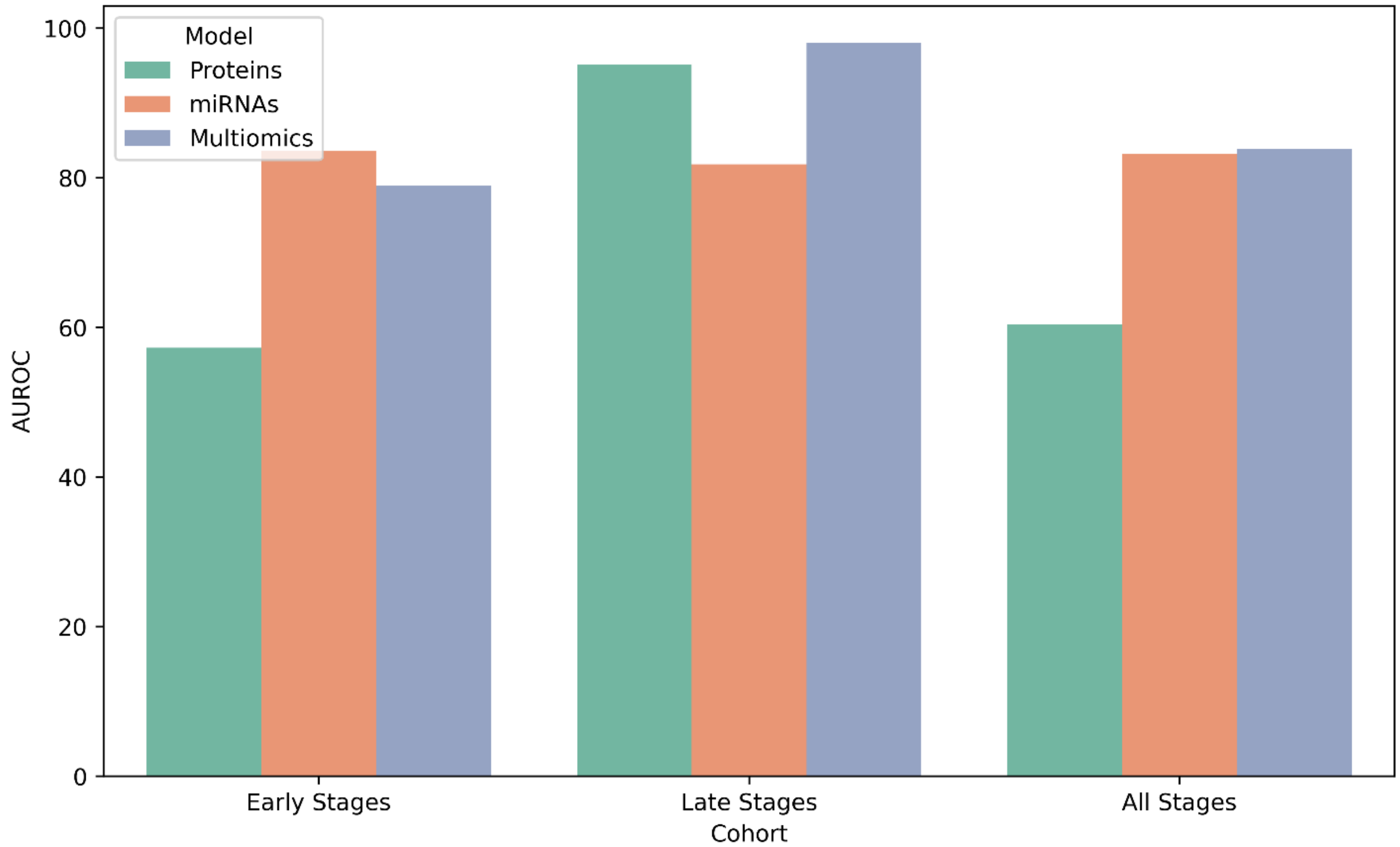

Supplementary Figure 2. Model Performance Stratified by Clinical Stage. Protein tumor markers demonstrated stronger predictive performance in patients with late-stage disease, whereas miRNA-based models performed better in patients with early-stage disease. By integrating both miRNA and protein tumor markers, the multi-omics model leveraged the complementary strengths of each modality, resulting in more balanced performance across different clinical stages.

References

1. Abdipourbozorgbaghi M, Vancura A, Radpour R, Haefliger S. Circulating miRNA panels as a novel non-invasive diagnostic, prognostic, and potential predictive biomarkers in non-small cell lung cancer (NSCLC). *Br J Cancer*. 2024;131(8):1350-1362.
2. Liao J, Shen J, Leng Q, et al. MicroRNA-based biomarkers for diagnosis of non-small cell lung cancer (NSCLC). *Thorac Cancer*. 2020;11(3):762-768.
3. Ying L, Du L, Zou R, et al. Development of a serum miRNA panel for detection of early stage non-small cell lung cancer. *Proc Natl Acad Sci U S A*. 2020;117(40):25036-25042.
