# Supplementary material for: Multi-Institutional Integration of Circulating miRNAs and Protein Tumor Markers for Early Lung Cancer Detection
Source: JTO Clin Res Rep. 2026 Jul 10;7(10):101051. doi: 10.1016/j.jtocrr.2026.101051 (PMC13584035; doi:10.1016/j.jtocrr.2026.101051)
Supplement: Supplementary file 2 [file mmc2.docx]

# Supplementary Materials 2

Institutional Review Board (IRB) Approvals for Participating Hospitals

| **Hospital** | **IRB Approval No.** | **Approval Date** | **Approval Period** |
| --- | --- | --- | --- |
| Fu Jen Catholic University Hospital | FJUH113357 | 2024-06-06 | 2024-06-06 to 2025-06-05 |
| Kaohsiung Chang Gung Memorial Hospital | 202400389A3 | 2024-05-05 | 2024-05-05 to 2025-04-24 |
| Kaohsiung Medical University Chung-Ho Memorial Hospital | KMUHIRB-F(I)-20240163 | 2024-05-17 | 2024-05-17 to 2024-11-16 |
| Keelung Chang Gung Memorial Hospital | 202400389A3 | 2024-05-05 | 2024-05-05 to 2025-04-24 |
| Linkou Chang Gung Memorial Hospital | 202400389A3 | 2024-05-05 | 2024-05-05 to 2025-04-24 |
| Mackay Memorial Hospital (Taipei) | 24CT008Ae | 2024-05-14 | 2024-05-14 to 2025-05-13 |
| Shin Kong Wu Ho-Su Memorial Hospital | 20240410D | 2024-06-13 | 2024-08-01 to 2025-07-31 |
| Taipei Medical University Hospital | N202407089 | 2024-08-07 | 2024-08-07 to 2025-08-06 |
| Tri-Service General Hospital | A202403001 | 2024-07-30 | 2024-07-30 to 2025-07-29 |
| New Taipei City Tucheng Hospital | 202400389A3 | 2024-05-05 | 2024-05-05 to 2025-04-24 |
| National Taiwan University Hospital Hsin-Chu Branch | 202407215DSA | 2024-09-06 | 2024-09-06 to 2025-09-05 |

*Kaohsiung Chang Gung Memorial Hospital, Keelung Chang Gung Memorial Hospital, Linkou Chang Gung Memorial Hospital, and New Taipei City Tucheng Hospital were reviewed and approved jointly under a single Chang Gung Medical Foundation Institutional Review Board protocol (202400389A3).*
